# Clinical application of genotypic co-receptor tropism testing from viral RNA and proviral DNA: week 24 analysis of the Berlin maraviroc cohort

**DOI:** 10.1186/1758-2652-13-S4-P129

**Published:** 2010-11-08

**Authors:** MJ Obermeier, A Carganico, S Dupke, D Schranz, C Cordes, M Freiwald, G Klausen, S Köppe, C Schuler, C Mayr, D Schleehauf, T Berg, I Krznaric, A Baumgarten

**Affiliations:** 1 MVZ mib AG, Labor Dr. Berg, Berlin, Germany; 2 MVZ mib AG, Praxis Driesener Strasse, Berlin, Germany; 3 MVZ mib AG, Berlin, Germany; 4 Praxis Schranz Fischer, Berlin, Germany; 5 MVZ -Praxis City Ost, Berlin, Germany; 6 Ärztezentrum Nollendorfplatz, Berlin, Germany; 7 Praxis Hintsche Klausen, Berlin, Germany; 8 Gemeinschaftspraxis Mehringdamm, Berlin, Germany; 9 Praxisgemeinschaft Turmstrasse, Berlin, Germany; 10 Ärzteforum Seestrasse, Berlin, Germany; 11 Praxiszentrum Kaiserdamm, Berlin, Germany

## Objectives

Before initiating an antiretroviral combination therapy which includes Maraviroc as one of its components, a coreceptor tropism test has to be performed. As Maraviroc is only effective against HIV strains that are using CCR5 as coreceptor a thoroughly validated and reliable assay should be performed. In case of treatment change due to toxicity, testing should also be possible using proviral DNA.

## Material and methods

Included in the study cohort of this non-interventional observational study are 157 patients. Only inclusion criteria was a treatment with Maraviroc and a previous tropism test. Phenotypic tropism tests were performed by Monogram biosciences (TROFILE®). For Genotypic tropism determination from V3 loop sequence data geno2pheno_[coreceptor]_ was used. Data acquisition for week 12 and week 24 is already finished. It is planned to collect data for 96 weeks for all the patients. Data analysed are besides viral load and the other components of the HAART, genotypic resistance assay at baseline, immunologic parameters and treatment side effects.

## Results

Genotypic tropism results were available for 88 patients from viral RNA sequences and for 53 patients from proviral DNA sequences. A TROFILE® result was available for 70 of the patients as 71 of the patients had a viral load below 1000 cop./ml and a testing with TROFILE® was not possible. In the intent to treat (ITT) analysis 70% of the patients were successfully treated at week 24, defined as a viral load decline of at least 3 logs or a viral load below detection limit of 50 copies/ml. The positive predictive value (PPV) of geno2pheno using a FPR cut-off of 10% was 77% (PPV TROFILE®: 66%). In the subgroup of patients where only a genotypic tropism test from proviral DNA was available 86% of the patients had a viral load below detection limit of 50 copies/ml (70% at baseline).

## Conclusions

On this clinical dataset performance in terms of predictive power is slightly better when using geno2pheno compared to TROFILE®. Therefore genotypic tropism testing proves to be an easy and inexpensive alternative to phenotypic tropism testing.

Especially in the group of patients, where only a genotypic tropism test could be performed from proviral DNA, a high rate of successful treatments was observed, showing that this method makes it possible to perform tropism testing guided treatment change despite undetectable viral load.

geno2pheno_[coreceptor]_: http://www.geno2pheno.org

